# Mediating role of coping styles on the relationship between personality types and mental disorders in cardiovascular patients: a cross-sectional study in Iran

**DOI:** 10.1186/s12888-023-04742-8

**Published:** 2023-04-07

**Authors:** Behzad Yaghoubi, Reza Nemati, Khosrow Agin, Ali Mohammad Beigi Dehaghi, Meysam Gazmeh, Farshad Rezaei, Elham Amirbandi, Akram Farhadi

**Affiliations:** 1 Loghman Hakim Hospital, Shahid Beheshti University of Medical Sciences, Tehran, Iran; 2 Department of Medical Emergencies, School of Allied Medical Sciences, Bushehr University of Medical Sciences, Bushehr, Iran; 3 Heart Center Hospital, Bushehr University of Medical Science, Bushehr, Iran; 4 Mehr Borazjan Hospital, Social Security Organization, Borazjan, Bushehr, Iran; 5 The Persian Gulf Tropical Medicine Research Center, The Persian Gulf Biomedical Sciences Research Institute, Bushehr University of Medical Sciences, Bushehr, Iran

**Keywords:** Personality types, Coping styles, Mental disorders, Million, NEO

## Abstract

**Background:**

Many mental problems lead to the occurrence of physical diseases, causing worse consequences of diseases. Despite many studies in the field of personality types and types of mental disorders, this relationship and the mediating role of coping styles in cardiovascular patients are still not well known. Therefore, the present study was conducted to investigate the mediating role of coping styles in the relationship between personality types and mental disorders in cardiovascular patients.

**Method:**

The present study is a cross-sectional study that was conducted on 114 cardiovascular patients at the heart center in Bushehr, Iran. The sampling method is simple random sampling. Demographic information form, MCMI-III questionnaire, NEO-FFI questionnaire, and Lazarus and Folkman coping styles questionnaire were used to collect data. Data were analyzed using SPSS 22 and Amos 24 software. Descriptive statistics methods (mean, variance and percentage), Pearson correlation, and structural equation model (SEM) were applied to analyze the data.

**Results:**

The findings showed that the two variables of personality types and problem-oriented explain 15.2% of the variable of mental disorders, of which 10.7% is related to the variable of personality types and 4.5% is related to the intermediate variable of problem-oriented. Among the personality types, the neurotic personality type has the biggest role (0.632) and has a direct and significant effect on mental disorders. Also, the personality types of extroversion (-0.460), agreeableness (-0.312), and responsibility (-0.986) exert inverse and significant effects on mental disorders.

**Conclusion:**

The results of the present study showed the frequency of personality disorders and other mental disorders among heart patients. Problem-oriented coping style plays a mediating role between personality types and mental disorders.

## Introduction

Mental health has drawn more attention by researchers during recent years. Mental illness refers to a wide range of different mental health disorders [1]. Psychological disorders are a group of disorders, which affect thinking and behavior, cause discomfort for the affected person or create weakness in him [1]. According to the latest reports of the World Health Organization in 2022, psychological disorders are very common in all countries. Mental disorders affect one person out of eight worldwide. The occurrence of various mental disorders differs by gender and age. Anxiety disorders and depressive disorders are the most prevalent among both men and women [2].

Mental illnesses can cause unpleasant complications in the overall health of a society. All over the world, mental illness at any age with any background can lead to suicide or the killing of a member of their family [2]. Furthermore, psychological disorders have significant economic consequences. Schizophrenia, in particular, is the most costly mental disorder for society when viewed through an economic lens [3]. Although depression and anxiety disorders may be less expensive for individuals to manage, their high prevalence means they contribute significantly to overall national costs [2]. Despite progress in some countries, people with mental illnesses often experience severe human rights violations, discrimination, and stigma [2]. The World Health Organization (WHO) emphasizes that increasing investment in all fields is needed to increase awareness of mental health, and reduce the stigma of the disease [4], because psychological disorders do not end only with mental and thinking disorders, but they are directly related to the occurrence of physical diseases [5]. The situation is particularly worrying concerning because mental disorders frequently co-occur with one or more chronic physical illnesses, resulting in more severe physical health outcomes for the individual. Additionally, individuals with mental disorders tend to receive less healthcare for their physical health conditions compared to those without mental disorders who have the same illnesses. According to reports, in low- and middle-income countries, roughly 80% of individuals with severe mental disorders face challenges accessing mental healthcare and do not receive treatment. This makes mental disorders the second leading cause of death in these nations [6]. Mental and physical health are closely intertwined, with multiple links between mental health and chronic physical conditions. These connections can significantly impact a person’s quality of life, healthcare needs, and demand for publicly funded services, and have wider consequences for society. According to the World Health Organization, health is not merely the absence of illness or infirmity but a state in which an individual’s physical, mental, and social well-being are all fully realized. The WHO emphasizes that mental health is a crucial component of overall health, stating that “there can be no health without mental health.“ [6].

The association between mental and physical health is nowhere more noticeable than in chronic diseases. Numerous studies have illustrated the connection between mental disorders such as depression, anxiety, schizophrenia, and bipolar disorders and chronic physical illnesses [7] such as cancer [8, 9], heart disease, stroke [10, 11], diabetes [12, 13], obesity [14] and chronic obstructive pulmonary disease (COPD) [15] Cardiovascular diseases are the leading cause of both mortality and morbidity on a global scale, among chronic illnesses [16]. According to the statistics revealed by WHO in 2019, cardiovascular diseases are the most common cause of death [16].

An increasing amount of evidence suggests that mental health is correlated with risk factors for heart disease, not only before the diagnosis of a mental disorder but also during treatment. The mechanisms responsible for these effects may be either direct, involving biological pathways, or indirect, through engaging in behaviors that heighten the risk of developing heart disease [17]. Individuals who suffer from chronic depression, anxiety, stress, and post-traumatic stress disorder (PTSD) may undergo specific physiological changes in their bodies, such as increased cardiac reactivity characterized by elevated blood pressure and heart rate, reduced blood flow to the heart, and prolonged high levels of cortisol. These alterations in bodily functions can result in the accumulation of calcium in arteries, metabolic disorders, and ultimately, heart disease [18–24]. Evidence indicates that mental health disorders, such as depression, anxiety, and PTSD, can develop following cardiac events, such as heart failure or heart attack [17, 25–27]. These disorders may emerge in the aftermath of an acute cardiac event, such as experiencing pain, fear of death or disability, and facing financial difficulties that are associated with the event [17, 25].

The odds of adopting behaviors such as smoking, a sedentary lifestyle, or not taking prescribed medications can be raised by mental health disorders such as anxiety and depression. This is because people with mental health disorders may possess less healthy coping mechanisms for managing stressful situations, and may find it challenging to adopt healthy lifestyle habits that can mitigate the risk of developing heart disease [17]. According to the latest statistics published by the American Heart Association, in 2020, approximately 19.1 million deaths worldwide were attributed to CVD. The age-adjusted death rate per 100,000 population was 239.8. The report considers the Middle East as one of the highest death rates due to CVD [28]. Research performed in southern Iran showed that the prevalence of CVD in this region is higher than in other regions and countries. According to their research, the frequency of cardiovascular disease (CVD) was 10.3%, meaning that there were around 10,300 instances of CVD per 100,000 individuals. A separate study on the worldwide impact of CVD revealed that the standardized prevalence of the disease exceeded 9,000 cases per 100,000 individuals in several countries, including Iran, Morocco, Oman, Zambia, and West Africa. Conversely, the standardized prevalence of CVD in Singapore, Japan, South Korea, Italy, Western Europe, and the United States was less than 5,000 per 100,000 individuals, which was lower in comparison to the estimated prevalence reported in the current study [29]. These reports can be the result of high-risk behaviors and lifestyles in these areas [29]. Personality types are among the things that determine the type of behavior and lifestyle and are directly related to the occurrence of mental disorders [30].

### Personality types and mental disorders

The examination of the function of personality in psychological trauma has been a prominent area of focus for a significant period. The five-factor model of personality (FFM), commonly referred to as the “Big Five” model, is the most popularly recognized model of personality [31]. The five fundamental personality traits of FFM include neuroticism, extraversion, openness, agreeableness, and conscientiousness. Neuroticism pertains to an individual’s proneness to emotional instability and sensitivity. Openness is indicative of an inclination towards novel experiences and creativity. Agreeableness and extraversion are personality traits that center around interpersonal relationships. Extraversion characterizes individuals who are sociable, enthusiastic, assertive, and seek excitement and happiness. Agreeableness describes individuals who are altruistic, kind, humble, trusting of others, and reliable. Conscientiousness is defined as the inclination to be meticulous, competent, and systematic. These five personality traits are regarded as the fundamental dimensions of personality [32]. Individuals with schizophrenia showed higher levels of neuroticism [33]. FFM personality traits can heighten susceptibility to psychotic disorders. Elevated levels of neuroticism denote a susceptibility to experiencing anxiety and distress, and this personality trait has been discovered to be a risk factor for developing schizophrenia [34–36], In contrast, a study has shown that increased levels of extraversion can diminish this risk [37].

According to reports, a limited number of personality traits, including optimism, conscientiousness, openness, and curiosity, are linked to favorable health outcomes in individuals with CVD, and are therefore deemed as cardio-protective personality traits [38]. Identified as a defensive factor against CAD risk, optimism is a positive personality trait characterized by the inclination to anticipate favorable outcomes [39].

Despite the studies mentioned affirming the positive correlation between psychological disorders and personality traits, as well as their impact on cardiovascular diseases, certain findings demonstrate that the approach and manner in which individuals confront life issues, problems, and events, as well as their use of diverse strategies, can have an effect in this regard [40, 41].

### The mediating role of coping style

The behavioral and cognitive efforts of individuals to address specific external and/or internal demands under stress is known as the coping style [42]. In general, coping styles are classified into two categories: problem-focused and emotion-focused coping. Problem-focused coping entails strategies for addressing issues that cause emotional distress, such as problem-solving, help-seeking, and cognitive restructuring. On the other hand, emotion-focused coping pertains to techniques that alleviate negative emotions, such as rumination and delusions [43]. The application of coping styles is also influenced by how individuals interpret stressors, and the outcomes of different coping styles can vary. Those who use problem-focused coping tend to find effective ways to manage challenging situations, leading to better adaptation [44], while individuals who adopt an emotion-focused coping style may tend to cope with their problems passively by avoiding them [45]. Research shows a positive relationship between coping styles and psychological disorders [46, 47]. According to a study by Leszko et al. (2020), emotion-focused coping strategies are more likely to be adopted by individuals with high levels of neuroticism, whereas those with high levels of conscientiousness are more likely to adopt problem-focused coping strategies [40]. Numerous studies have confirmed the direct correlation between personality traits and coping styles [41, 46–50].

A study conducted by Svensson et al. (2016) revealed that in a general population without any health issues, using problem-focused coping strategies was significantly linked to decreased mortality rates from cardiovascular diseases. According to Svensson et al. (2016), in a healthy general population, the use of problem-focused coping strategies was significantly associated with a reduced risk of mortality from cardiovascular diseases. The researchers proposed that this finding could be due to the indirect effect of coping strategies on risk factors for CVD. Therefore, they suggested that educating individuals on stress management and coping strategies may lead to improved lifestyle habits, increased participation in screening programs, and better adherence to treatment plans [51]. Sadr Bafghi et al. (2018) investigating the use of coping styles in patients with MI and healthy people without a history of MI, concluded that 53.6% of patients with heart attacks employed an emotion-focused coping style, and 12.7% of them used avoidance coping style. And only 33.6% of patients used a problem-focused coping style. This is while 63.6% of healthy people without a history of heart attack used a problem-focused coping style [52]. Therefore, based on these studies, heart patients more often use emotion-focused coping strategies, such as suppressing negative thoughts or feelings, praying, overeating, drinking alcohol, using drugs, releasing suppressed emotions, meditation, blaming, denial, and seeking social support [52]. This is despite the fact that most of the proposed methods of emotion-focused coping strategies are cardiovascular risk factors that can result in a heart attack [53].

### The present study

**Fig. 1:**
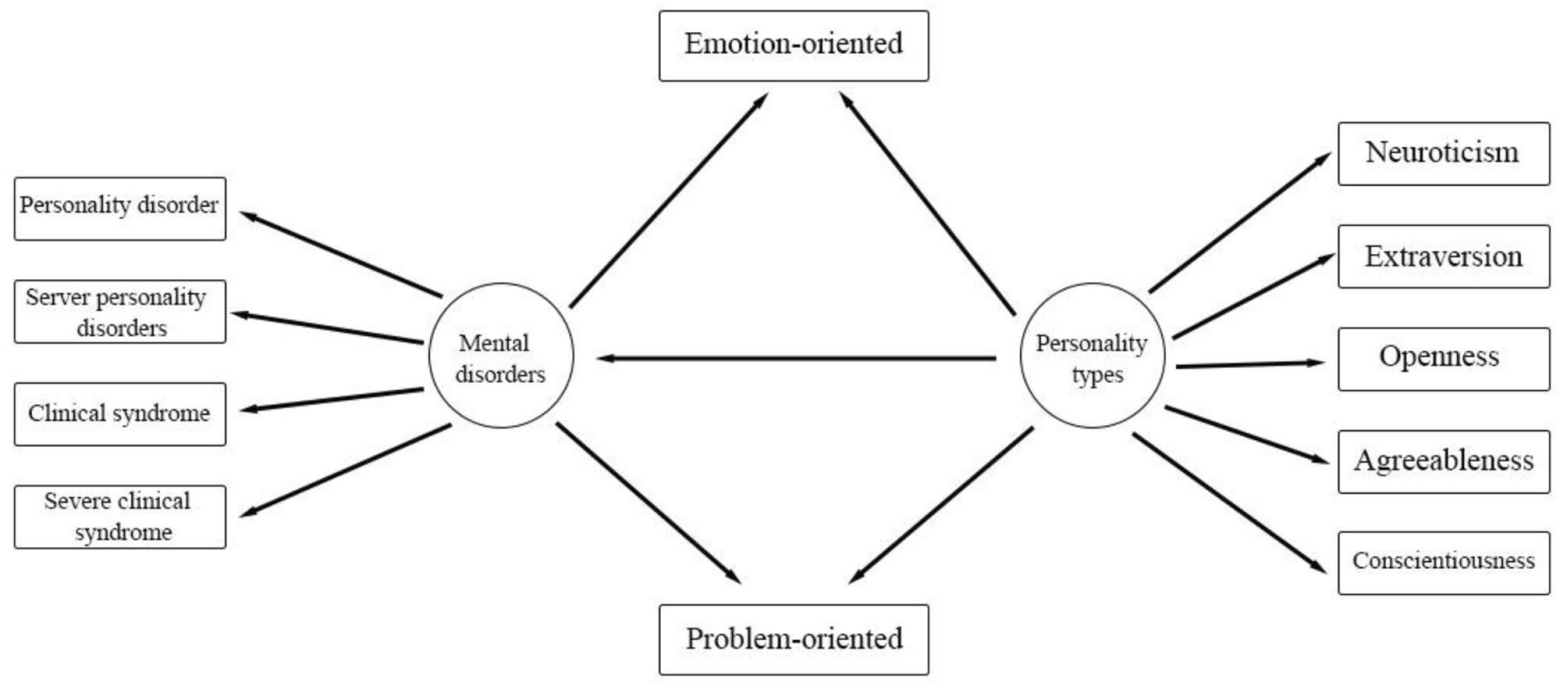
The supposed model

The purpose of this study was to investigate the mediating role of coping styles in the relationship between personality types and mental disorders in cardiovascular patients. Although previous studies have provided evidence of the impact of personality traits and psychological disorders, and the causal relationship between personality traits and coping styles, there are still several unanswered questions. To the best of our knowledge, no research has specifically focused on the mediating role of coping styles in the relationship between personality traits and psychological disorders in cardiovascular patients. Therefore, this study aimed to examine the mediating effects of coping styles on personality traits and psychological disorders According to our knowledge, this is the first comprehensive experimental study that includes a specialized examination of personality traits, types of psychological disorders, and coping styles among cardiovascular patients in southern Iran. Based on the proposed model (Fig. 1), we assume that different types of coping styles strengthen the relationship between personality types and psychological disorders.

## Methods

### Design, settings, and participants

The present study is a cross-sectional study and the research population is all patients hospitalized in Bushehr Heart Center (North of the Persian Gulf). This study was conducted from 22 to 2019 to 17 February 2020. Sampling was done as a simple random sampling among hospitalized patients based on the patient information registration system in the hospital. The inclusion criteria were based on the diagnosis of a specialist doctor, a patient with ACS or MI, and the patient’s consent to participate in the study. In addition, the concept application criteria of the Million-III Questionnaire for the Iranian society emphasizes that the participants must be over 18 years old and have at least a middle school certificate [54], which was added to the inclusion criteria for this study. The entire research includes ACS and MI patients hospitalized in CCU, whose monthly number is about 120 people, and in two months, which is the time frame of the study, it is approximately 240 people.

To determine the sample size, considering the type of research (descriptive), the reduction of errors, the representation of members of the community, and according to the study of Aluja et al. (2007) [55], 101 people were calculated, which was considered 117 people with 15% reduction.

Sample volume with formula

$$n=\frac{N\times {Z}_{\frac{\alpha }{2}}^{2}\times {\sigma }^{2}}{{\epsilon }^{2}\left(N-1\right)+{Z}_{\frac{\alpha }{2}}^{2}\times {\sigma }^{2}}$$

It has been extracted that the level of accuracy is ε = 0.7, the standard deviation is σ = 4.7, and the test error level is α = 0.05.

### Data collection procedure and instruments

A demographic information collection form, MCMI-III questionnaire, NEO-FFI questionnaire, and coping styles questionnaire were used to collect data. In addition, the questionnaires were given to the patients when they did not have chest pain and not during the rounds of doctors and nurses or during medical care. Patients were kept calm while answering the questions.

The demographic information form included age, gender, level of education, marital status, employment status, income, city of residence, disease diagnosis, length of hospitalization, history of cardiovascular diseases, cardiac risk factors, and history of known mental illnesses. All this information was collected by self-report.

#### Millon clinical multiaxial inventory, third edition (MCMI-III)

The MCMI-III questionnaire was designed by Theodore Millon in 1944 [56]. This questionnaire has 175 yes-no questions. This questionnaire has 24 clinical scales and 4 validity indicators. These 24 scales are divided into 4 general categories [57]. The clinical patterns of personality have 11 subgroups, including schizoid personality, avoidant personality, depressed personality, dependent personality, dramatic personality, narcissistic personality, antisocial personality, abusive personality, compulsive personality, negative personality, and narcissistic personality. Severe clinical patterns of personality include 3 subgroups; schizotypal personality, borderline personality, and paranoid personality. There are 7 clinical syndromes including anxiety disorder, somatic disorder, bipolar disorder, depressive disorder, alcohol dependence, substance dependence, and post-traumatic stress disorder. The severe clinical syndrome has 3 subcategories including thought disorder, major depression, and delusional disorder [57]. The analysis of this article is also based on this classification. To use this tool, participants must be over 18 years old and have at least a middle school certificate [54].

In clinical personalities and severe personality pathology, the Millon questionnaire classifies patients into 3 groups; “likely to possess traits of the construct”, “clinically significant personality trait” and “personality disorder”. It categorizes clinical syndromes and severe clinical syndromes into three groups: “likely to have some symptoms of the syndrome,“ “presence of the syndrome,“ and “prominence of the syndrome” [58]. The validity of this questionnaire was obtained by the test-retest method in the patient group (0.793 − 0.611), and in the non-patient group (0.972 − 0.795), and the Cronbach’s alpha reliability was 0.89 − 0.64 [54].

#### NEO five-factor inventory (NEO-FFI)

The NEO-FFI is a tool developed by Costa and McCrae (1988) that evaluates the five personality traits of neuroticism, extraversion, openness, conscientiousness, and agreeableness through 60 items [59]. Respondents rate each item on a five-point Likert scale ranging from completely disagree = 0 to completely agree = 4. To assess its reliability and validity, Ziapour et al. (2015) computed Cronbach’s alpha values for neuroticism, extraversion, agreeableness, openness, and conscientiousness, which were 0.91, 0.78, 0.76, 0.73, and 0.86, respectively [60].

#### Coping styles questionnaire (CSQ)

This questionnaire was designed by Lazarus and Folkman in 1980 [61]. The questionnaire used in the present study is the revised version by Lazarus and Folkman (1985), containing 66 questions that are scored on a 4-point Likert scale. The coping strategies questionnaire has 8 subscales, which are: (1) direct coping; (2) avoiding or distancing; (3) restraint; (4) seeking social support; (5) Responsibility; (6) escape-avoidance; (7) planned problem-solving; (8) Positive reassessment. These 8 coping patterns are divided into two general categories: problem-oriented strategies and emotion-oriented strategies. Problem-oriented strategies include seeking social support, taking responsibility, planned problem-solving, and positive reappraisal. Emotion-oriented strategies include direct confrontation, avoidance, escape-avoidance, and self-restraint [62, 63]. The validity and reliability of this questionnaire were confirmed in the study of Ahadi et al. (2014) in Iranian society [64].

### Ethical consideration

This study was reviewed and approved by the ethics committee of Bushehr University of Medical Sciences and has the code of ethics “IR.BPUMS.REC.1400.135”. It was explained to the participants that participating in the research is optional and due to the confidentiality of information, their names and surnames will not be included in the questionnaire. In addition, informed consent was obtained from the participants based on the Helsinki Treaty.

### Data analysis

Data analysis was done by the Statistical Package for the Social Sciences (SPSS) version 22 (SPSS Inc., Chicago, IL, USA) and Amos24 software. Descriptive statistics methods (mean, variance, and percentage) were used for describing socio-demographic characteristics, average coping styles of patients participating, and frequency of psychological disorders among study participants. Pearson correlation was used to evaluate the correlation between coping styles and psychological disorders. SEM was used to test the hypothesized interrelationships among personality types, coping styles, and mental disorders. The methods used to conduct SEM are model specification, model identification, model estimation, and model testing. The structural model was specified by developing hypotheses regarding the relationships among the variables based on previous research and theory. In this model, in particular, the mediating role of coping styles between the two variables of personality types with mental disorders has been investigated. In SEM analysis, resampling (bootstrap method with 2000 iterations) was applied. The goodness of fit indices was used to assess the model fit, which involved examining the direction, magnitude, and significance of parameter estimates. The study examined two types of effects: (a) direct effects, which refers to direct relationships between variables, and (b) indirect effects, which refers to the relationships that are mediated through intervening variables.

## Result

### Sociodemographic characteristics

Out of 120 people who participated in the study, 6 questionnaires were excluded due to incomplete completion, and finally, the questionnaires of 114 patients were included in the study (Response rate = 95%). Table 1 shows the participants’ demographic specifications.

**Table 1:** Demographic characteristics of patients participating in the study (n = 114)

Variables	Subgroup	Number (%)
**Age**	under 40 years	28 (24.6)
	over 40 years old	86 (75.4)
**Gender**	Man	93 (81.6)
	Woman	21 (18.4)
**Education**	Middle school	25 (21.9)
	Diploma	55 (58.2)
	Bachelor and above	34 (29.8)
**Marital status**	Single	22 (19.3)
	Married	92 (80.7)
**Employment status**	Unemployed	20 (17.5)
	Employed	85 (74.6)
	Retired	9 (7.9)
**Income**	Less than $100	4 (3.5)
	100 to $300	17 (14.9)
	$ 300 to $500	51 (44.7)
	Over $ 500	42 (36.8)
**Cardiovascular risk factors**	Yes	102 (89.5)
	No	12 (10.5)
**History of heart disease**	Yes	15 (13.2)
	No	99 (86.8)
**History of psychiatric illness**	Yes	4 (3.5)
	No	110 (96.5)

### Average coping styles of patients participating in the study

The findings of the study showed that among coping styles, the emotion-oriented coping style (35.00 ± 11.11) has a higher mean than the problem-oriented coping style (33.35 ± 9.79).

### Frequency of psychological disorder among study participants

Results showed that with BR 70–74, the most frequent personality disorders were depressive, histrionic, passive-aggressive, and schizoid. The most frequent severe personality pathology was schizotypal. With BR 75–84, the most frequent personality disorders were again depressive and histrionic and severe personality pathology was borderline. With BR 85 and above, the most frequent personality disorders were dependent and passive-aggressive, and severe personality pathology was paranoid. (Table 2)

When using BR 75–84, the moderate clinical syndromes that appeared most frequently were somatoform and bipolar, and all severe syndrome scales were equally prevalent. On the other hand, when using BR 85 and above, the most common moderate clinical syndromes were drug dependence, anxiety, and bipolar, while the severe syndrome scale was thought disorder. (Table 2)

**Table 2:** Frequency of personality disorders in patients participating in the study (n = 114)

Mental disorders		likely to possess traits of the construct (%)	clinically significant personality trait (%)	personality disorder
**Clinical personalities**				
**1**	Schizoid	7.3	3.6	2.4
**2 A**	Avoidant	4.8	6	0
**2B**	Depressive	12.1	9.7	1.2
**3**	Dependent	2.4	2.4	6
**4**	Histrionic	10.9	9.7	2.4
**5**	Narcissistic	3.6	1.2	0
**6 A**	Antisocial	6	0	0
**6B**	Aggressive(sadistic)	0	3.6	0
**7**	compulsive	4.8	3.6	1.2
**8 A**	Passive-aggressive (Negativistic)	7.3	3.6	3.6
**8B**	Self-Defeating	6	3.6	2.4
**Severe personality pathology**				
**s**	Schizotypal	3.6	1.2	0
**c**	Borderline	2.4	3.6	0
**p**	Paranoid	2.4	1.2	2.4
**Clinical syndromes**				
**A**	Anxiety	32.9	6	2.4
**H**	Somatoform	41.4	9.7	1.2
**N**	Bipolar: manic	30.4	8.5	2.4
**D**	Dysthymia	40.2	1.2	1.2
**B**	Alcohol dependence	29.2	0	0
**T**	drug dependence	30.4	6	3.6
**R**	PTSD	24.3	1.2	0
**Severe clinical syndromes**				
**SS**	thought disorder	46.3	2.4	1.2
**CC**	major depression	39	2.4	0
**PP**	Delusional disorder	26.8	2.4	0

### Average personality types and personality disorders and correlation between them in patients participating in the study

Among the variables of personality types, neuroticism had a negative and significant correlation with responsibility (P value < 0.01), agreeableness (P value = 0.02) and extroversion (P value = 0.009). Neuroticism showed a positive and significant correlation with personality disorders (P value = 0.007) and clinical symptoms (P value = 0.02). Extroversion personality type had a positive and significant relationship with responsibility (P value < 0.01). There was a positive and noticeable significant relationship between openness with severe personality disorders (P value = 0.02) and clinical symptoms (P value = 0.03) (Table 3).

Agreeable personality type showed a significantly positive relationship with responsibility (P value = 0.001), and significantly negative relationship with personality disorders (P value = 0.03), severe personality disorders (P value = 0.02), and severe symptoms (P value = 0.04). There was a negative significant association between the responsible personality type with personality disorders (P value < 0.01), severe personality disorders (P value = 0.03), clinical symptoms (P value = 0.007), and severe clinical symptoms (P value = 0.009). (Table 3).

**Table 3:** Average personality types and personality disorders and correlation between them in patients participating in the study (n = 114)

Variable	Mean ± SD	Neuroticism	Extraversion	Openness	Agreeableness	Conscientiousness	Personality disorder	Severe personality disorders	Clinical syndrome	Severe clinical syndrome
Neuroticism	20.06 ± 6.52	1								
Extraversion	28.21 ± 4.06	**-0.24** ^**^	1							
Openness	23.49 ± 3.60	0.09	0.05	1						
Agreeableness	31.19 ± 3.73	**-0.21** ^*^	0.09	0.06	1					
Conscientiousness	29.64 ± 5.72	**-0.62** ^**^	**0.45** ^**^	-0.08	**0.30** ^**^	1				
Personality disorder	48.37 ± 8.81	**0.25** ^**^	-0.09	0.15	**-0.19** ^*^	**-0.33** ^**^	1			
Severe personality disorders	47.72 ± 9.72	0.11	-0.05	0.20	**-0.21** ^*^	**-0.20** ^*^	**0.73** ^**^	1		
Clinical syndrome	46.55 ± 9.97	**0.20** ^*^	-0.02	**0.02** ^*^	-0.06	**-0.25** ^**^	**0.74** ^**^	**0.55** ^**^	1	
Severe clinical syndrome	45.19 ± 13.07	0.13	-0.1	0.11	**-0.19** ^*^	**-0.24** ^**^	**0.81** ^**^	**0.66** ^**^	**0.62** ^**^	1

**. Correlation is significant at the 0.01 level

*. Correlation is significant at the 0.05 level

### Investigating the mediating role of coping styles in the relationship between personality types and mental disorders in patients participating in the study

**Fig. 2:**
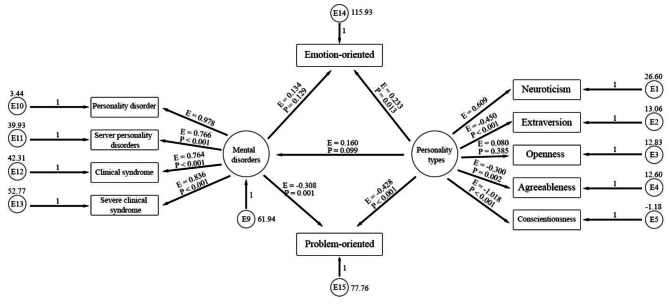
The first model: the mediation of problem-oriented and emotion-oriented coping styles in the relationship between personality types and mental disorders. (E = Estimate (Standardized values of regression coefficients), P = P value)

**Fig. 3:**
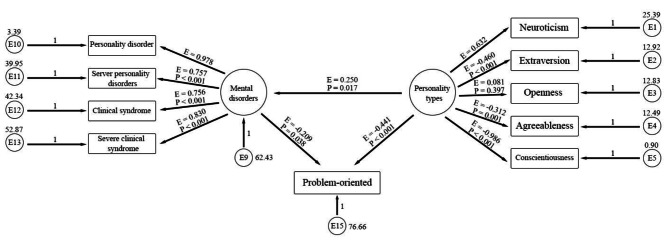
The second model: mediation of problem-oriented coping style in the relationship between personality types and mental disorders. (E = Estimate (Standardized values of regression coefficients), P = P value)

In order to investigate the mediating role of problem-oriented and emotion-oriented coping styles, between personality types and mental disorders in the first model (Fig. 2) with the Sobel test, regression coefficients and standard deviation were used. The results of the Sobel test showed that the mediating role of the excitatory variable was not accepted (Z = 1.300, P = 193). While the mediating role of the problem-oriented variable was confirmed using the Sobel test (Z = 2.597, P = 009) (Table 4). Therefore, the emotional coping style was removed from the conceptual model under study, and the implementation model (Fig. 3), and its indicators were reported again. (Model 2, Table 4)

**Table 4:** Goodness of fit indexes of structural equation models

Type of goodness of fit index	Index limits for acceptable fit	GFI of the first model	GFI of the second model
Statistical value (df)X^2^	The ratio of the X^2^ statistic to the degree of freedom is < 5	149.049(41)	30.024(33)
P-value of X^2^ test		P < 0.05	P = 0.616
The ratio of the X^2^ statistic to the degree of freedom		3.636	0.910
**RMSEA**	< 0.08	0.153	0.000
**CFI**	> 0.90	0.799	0.998
**NFI**	> 0.90	0.749	0.936
**GFI**	> 0.85	0.857	0.951
**AGFI**	> 0.85	0.799	0.919

RMSEA: Root Mean Square Error of Approximation

CFI: Comparative Fit Index

NFI: Normed Fit Index

GFI: Goodness Fit Index

AGFI: Adjusted Goodness of Fit Index

According to the values related to goodness of fit indices, model number 2 is at a good and acceptable level in terms of all goodness of fit indices.

Table 5 shows the results of the estimation of regression coefficients and their significance levels. The pattern related to the variables of this model includes the dependent variable (personality types), mediator variable (problem-oriented), and dependent variable (mental disorders). According to the findings, all relationships between the personality type variable and the mental disorder variable were significant when the problem-oriented mediating variable was present as a mediator. Additionally, there was a significant direct effect of the personality types variable on the problem-oriented mediating variable, as well as a significant direct effect of the problem-oriented mediating variable on the mental disorder variable. The indirect effects were estimated using the bootstrap method.

**Table 5:** Estimation of regression coefficients of model 2

Relationships between variables	Estimate	S. E	Standardized	P
**Personality type** → **Mental disorders**	0.523	0.219	0.250	0.017
**Personality type** → **Problem-oriented**	-1.048	0.238	-0.441	< 0.001
**Problem-oriented** → **Mental disorders**	− 0.184	0.088	-0.209	0.038
**Personality type** → **Problem-oriented** → **Mental disorders**	0.19	0.093		< 0.001
**Personality type** → **Neuroticism**	1.000		0.632	< 0.001
**Personality type** → **Extraversion**	-0.453	0.099	-0.460	< 0.001
**Personality type** → **Openness**	0.071	0.084	0.081	0.397
**Personality type** → **Agreeableness**	-0.283	0.089	-0.312	0.001
**Personality type** → **Conscientiousness**	-1.368	0.231	-0.986	< 0.001
**Mental disorders** → **Personality disorder**	1.000		0.978	< 0.001
**Mental disorders** → **Severe personality disorders**	0.855	0.078	0.757	< 0.001
**Mental disorders** → **Clinical syndrome**	0.875	0.080	0.756	< 0.001
**Mental disorders** → **Severe clinical syndrome**	1.259	0.095	0.830	< 0.001

The findings showed that among the personality types, the neurotic personality type has the highest role (0.632) and has a direct and significant effect on mental disorders. Also, the personality types of extroversion (-0.460), agreeableness (-0.312), and responsibility (-0.986) exert inverse and significant effects on mental disorders. Subgroups of mental disorders exerted direct and significant effects. The highest effect was related to personality disorders (0.978) (Table 5).

**Fig. 4:**
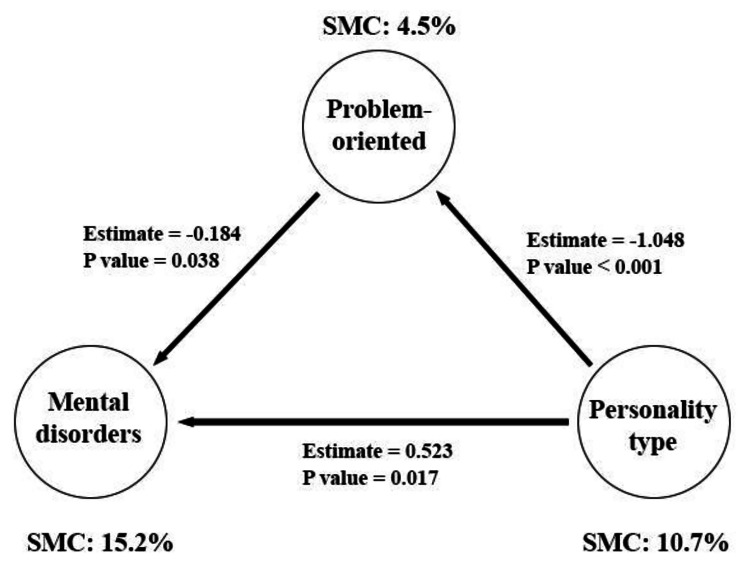
The third and final model: the relationship between the variable of personality types on the variable of mental disorders with the mediation of problem-oriented coping style

In addition, the two variables of personality types and problem-oriented explain 15.2% of the variable of mental disorders, of which 10.7% is related to the variable of personality types and 4.5% is related to the intermediate variable of problem-oriented. Thus, the final model was modified as number 3 (Fig. 4).

## Discussion

The first step to planning treatment in an area is to know its epidemiological aspects and then to have a good diagnostic method. The main reason for our decision to conduct this study was to search for the need for positive psychological intervention based on the prevalence of psychological problems and mediating variables in CHD patients. Therefore, the aim of this study was to investigate the mediating role of coping styles in the relationship between personality types and mental disorders in cardiovascular patients.

### Coping styles

The present study showed that in their personal life, patients use emotion-oriented coping styles such as direct confrontation, avoidance, escape-avoidance, and self-restraint in dealing with various events. In explaining this finding, it can be stated that considering that the emotion-oriented coping style only causes relief and temporary forgetting of problems, a person may face the stress of this problem again, causing an increase in cardiovascular problems [65]. The results of research by Svensson et al. (2016) showed that emotional coping styles are associated with an increased risk of cardiovascular disease mortality. Also, problem-oriented coping style and proactive dealing with stress sources were associated with a significant reduction in the incidence of stroke and heart attack mortality [51]. Fluharty et al. (2021) found that more use of emotion-focused coping is associated with less mental health [66]. Burns et al. (2016) reported in a study conducted on people with type 2 diabetes that only emotion-oriented coping predicted the likelihood of major depression [67]. On the other hand, Casagrande et al. (2019) showed that patients with high blood pressure and heart diseases use less appropriate coping strategies [68]. The results of these studies confirm the results of the present study.

During the acute phase of CVDs, dysfunctional types of emotion-focused coping strategies cause higher levels of distress when compared with patients who adopt problem-focused coping strategies [69]. Previous studies have shown that using emotion-focused coping strategies in stressful events is associated with a higher level of psychological distress, in contrast to problem-focused coping which is associated with lower psychological distress [70]. In our study, patients tend to adopt emotion-focused coping which means higher distress and worse outcome of treatment. Disruptive coping with stressful events causes problems like depression, anxiety, alcohol abuse, and hostility, all of which cause the worsening of the CHD patient’s condition [71].

### Frequency of mental disorders

MCMI-III revealed that above one-fourth of the study sample were likely to possess some symptoms of the syndrome, in moderate clinical syndromes and severe clinical syndromes which is a huge and impressive number to be found. This finding alarms every one of us to search for methods for evaluating the psychological health of patients with CVDs. Furthermore, about 10% of the study population had depressive and Histrionic personality traits and again, it is considered a high number of patients. In previous studies cluster B PDs (Antisocial, Borderline, Histrionic, and Narcissistic) were considered an independent risk factor for incident CVD in a community [72]. Considering BR 75 and above in MCMI-III as significant personality traits and disorders together, about 16.9% of the population had at least one of the cluster B PDs. 12.1% of the patients had Histrionic personality, which seems to be the most concerning matter among cluster B traits in an Iranian CVD sample. 3.6% of the patients had borderline personalities, and 2.4% were likely to have traits of the construct. Several factors were suggested for the relationship between PDs and CVDs. Among them are higher hostility and anger which are triggers of myocardial infarction, and behavioral aspects of them which suggest poorer control of CVD risk factors such as hypercholesterolemia, hypertension, and diabetes. And also, the importance of maintaining a good diet and healthy lifestyle in a CVD patient is compromised by patients with cluster B PDs [71, 73–75]. Previous studies have suggested that identification and treatment of Cluster B PD traits lead to better long-term treatment outcomes [72].

### Correlation between personality types and psychological disorders

The study’s findings indicate a positive correlation between neuroticism and personality disorders and clinical symptoms. Previous studies have suggested that people with high neuroticism tend to experience negative emotions such as anxiety, irritability, emotional instability, and depression [76, 77]. People who exhibit high levels of neuroticism often have a negative response to environmental stressors, perceive typical situations as ominous, and may exaggerate the impact of minor disappointments [78, 79]. These results show the positive relationship between neuroticism and personality disorders and clinical symptoms and confirm the results of the present study.

On the other hand, CVD patients need long-term treatments, and sticking to treatment plans is necessary. Treatment adherence is a serious issue in CVD patients because it directly affects morbidities, and lower adherence had been reported in patients with psychological problems [80]. One other thing that we need to consider is a treatment plan after detecting such problems. These kinds of treatment plans depend on the type of problems and a specific method cannot be applied to everyone due to the wide range of psychological problems. Zanarini et al. (2003) in a 6-year follow up suggested that in patients with borderline personality, treated patients were less likely to become obese, smoke cigarettes, and drink alcohol while they were more likely to exercise [81]. This single study highlights the importance of the treatment of a PD trait in CVD patients’ treatment outcomes.

### The mediating role of coping styles in the relationship between personality types and mental disorders

The results of the present study showed that the neurotic personality type exerts a direct and significant effect on the variable of mental disorders. The personality types of extroversion, agreeableness, and responsibility have significant and opposite effects on mental disorders. Some of the results of the study by Shi et al. (2018) confirm the results of the present study. Shi et al. reported in their study that the personality types of extraversion, openness, agreeableness, and conscientiousness significantly reduced psychotic disorders. Also, an increase in neuroticism and a decrease in extroversion, agreeableness, openness, and a decrease in conscientiousness can be related to psychotic disorders [30].

Such personality patterns may partly reflect structural differences in people’s affect, cognition, and behavior, which may provoke high levels of stress, as well as cause social isolation and reduce the chances of patient identification and treatment. Of course, in the current study, the effect of openness on mental disorders was not significant. Therefore, in this sense, it is inconsistent with the results of the present study. The reason for this difference can be considered the questionnaire applied to measure mental disorders. In Shi et al.‘s study, only the 16-question prodromal questionnaire was used. This questionnaire only evaluates the symptoms of psychotic disorder, including hallucinations and perceptual abnormalities, delusional thoughts, paranoia, and the content of abnormal thinking and negative symptoms [82]. However, in the current study, mental disorders were measured by Millon’s questionnaire, which covers a wide range of mental disorders. Therefore, the sum of the scores of other disorders can influence the effect of the variables.

On the other hand, the study by Boyette et al. (2013) reported a positive relationship between openness and psychotic symptoms [83]. These results are also in contrast with the results of the present study. The reason for this inconsistency can be the study population. The research samples in the current study were cardiovascular patients, and more than 96% of the patients had no history of psychological disorders as self-reported. Moreover, studies reported that people with cardiovascular disorders clearly have a low level of openness [84, 85]. This is because openness is related to cardiovascular hemodynamic response pathways and context in facing stress. It seems that people having a high level of openness are able to respond and adapt to new stress experiences [84]. Boyette et al. conducted a study on individuals with psychotic disorders and their families, as well as healthy individuals. The research findings suggested that people with a high level of openness personality trait tend to have broad and deep cognitive content and diverse life experiences. This openness and the ability to recognize various emotional situations often make these people optimistic and enable them to cope effectively with negative emotions [86]. Furthermore, patients with psychosis exhibit changes in their personality traits, including openness, during different stages of psychosis [30].

Previous studies have also explored the mediating role of coping styles. For example, Wilski et al. (2019) examined the locus of control over health and mental health in patients with multiple sclerosis, as well as the mediating effect of coping strategies. The study found that coping strategies could act as a mediator between health locus of control and mental health in patients with MS [87]. Zhang et al. (2019) explored the impact of psychological capital and occupational stress on teacher burnout, with a specific focus on the mediating role of coping styles. The study findings indicated that coping style played a significant mediating role in the relationship between job stress and teacher burnout [88]. Su et al. (2018) examined how coping styles mediate the relationship between personality and mental health in Chinese empty nesters. The findings indicated that coping styles played a mediating role in this relationship for the aging population [89]. The results of their study are in line with the results of the present study. It should be noted that in Su et al.‘s research, the Eysenck personality questionnaire was used to evaluate personality traits, which evaluates only three personalities, including extraversion, neuroticism and psychoticism, which includes 12 items. Also, to measure mental health, the mental health questionnaire of the elderly has been used, which examines five dimensions of ego, emotion, adaptive capacity, interpersonal communication, and cognitive function. In all three recent studies, Wilsky et al., Zhang et al., and Su et al., used personality or mental disorder assessment instruments that assessed general scales of mental health and did not address specifics [87–89]. This is while the current research uses the NEO-FFI questionnaire to examine personality traits, which examines five personalities. Also, the MCMI-III questionnaire was used to investigate mental disorders; as mentioned, this questionnaire evaluates mental disorders in more detail and measures 24 mental disorders. Therefore, this research can provide more accurate results than previous research.

The results of the present study showed that personality types have a causal relationship with mental disorders. This is while the mediator variable of problem-oriented coping style makes this relationship stronger by more than 15%. These results mean that people who use a less problem-oriented approach experience more psychological disorders. Such results can be explained due to the fact that the neurotic personality type had the greatest impact on psychological disorders and the fact that people with a neurotic personality type are pessimistic towards any strategy to deal with and regulate their emotions [30], as well as the fact that problem-oriented coping style focuses on dreaming about problems.

### Strengths, limitations, and suggestions

This is the first study on the mediating role of coping styles between personality types and personality disorders (using Millon’s questionnaire). One of the most important strengths of this study was applying different questionnaires to detect psychological problems simultaneously in every patient. Every questionnaire has its strengths and weaknesses. So, by using three different questionnaires, we tried to limit the chance of missing any potential psychological trait in cardiac patients. This study emphasizes the importance of personality traits and mental disorders in cardiovascular patients and provides evidence that relates the mediating role of coping styles to personality traits and mental disorders. On the other hand, the current study has carefully examined the extent and details of mental disorders in patients with heart disease, which can be very valuable from a clinical and practical point of view, and the special care of people with a mental health condition should be considered for this category of patients.

This study had also limitations. The present study was conducted in cardiovascular patients which limits the generalizability to some extent. One of the main reasons was halting sampling after the emergence of the novel Coronavirus (SARS-CoV-2) which interfered with the stress level of the patients. It also lowered access to the patients due to safety issues. The patients were suffering from CHD and they were in the acute phase of the disease. Patients who are not feeling well may report more personality pathology. In addition, due to the relatively high number of questions in the questionnaires, it was difficult to satisfy the patients to cooperate. Another limitation was the absence of a control group in the study; so that two populations of cardiovascular patients and healthy populations can be compared. Finally, this study was a cross-sectional design. Therefore, it does not lead to reasoning and causality.

It is suggested that in future studies, the mediating role of coping styles between personality traits and mental disorders should be investigated in longitudinal studies with a control group in order to achieve a deep understanding of this causality and relationships.

## Conclusion

The results of the present study showed the frequency of personality disorders and other mental disorders among heart patients. Also, the problem-oriented coping style has a mediating role between personality types and mental disorders.

It is worth mentioning that the patients may be normal and without any psychological problems, but personalities may change during a course of a medical illness [90, 91]. So, we suggest that even with no history and sign of a psychological problem in a previously healthy patient, they may develop psychological problems and as we mentioned, it affects morbidity and the final results of our treatments.

We believe that psychological problems in CVD patients have three axes; first as a risk factor for developing it, second as an exacerbating factor while confronting an acute cardiac problem, and third, as a confounding factor in recovering phase. So, trying to control psychological problems is strictly bound to controlling CVD. High rates of psychological problems in society and CVD patients make us double-think about their presence and search for it. Using MCMI-III or any other scale is not the point. The main point is that we should search for it and try to amend it, pre-think about it, and not postpone searching for it in course of the patient’s treatment. As a cardiologist searches for other risk factors like hypertension and tries to treat it, searching for and treating psychological problems should be a part of every cardiac patient. Having a psychological problem is an independent risk factor for developing CVDs, besides, the possibility of developing new psychological problems during the course of CVD, and also the high frequency of their presence persuades us to search for it and try to treat it like any other risk factor such as hypertension. Search for finding a proper early intervention such as cognitive behavioral therapy, social skills training, etc. should be performed in the future.

## Data Availability

The datasets used and/or analyzed during the current study are available from the corresponding author on reasonable request.

## List of abbreviations

MCMI: Millon Clinical Multiaxial Inventory
NEO: Neuroticism, Extraversion, Openness
SEM: Structural Equation Model
CVD: Cardiovascular Diseases
PD: Personality Disorder
DSM: Diagnostic and Statistical Manual of Mental Disorders
WCQ: Ways of Coping Questionnaire
MI: Myocardial infarction
ACS: Acute Coronary Syndrome
MI: Myocardial Infarction
CCU: Coronary Care Unit
BR: Base Rate
PTSD: Post-traumatic stress disorder
RMSEA: Root Mean Square Error of Approximation
CFI: Comparative Fit Index
NFI: Normed Fit Index
GFI: Goodness Fit Index
AGFI: Adjusted Goodness of Fit Index
SARS-CoV-2: Severe Acute Respiratory Syndrome Coronavirus 2
CHD: Congenital Heart Defect
